# Chimeric lipoproteins for leptospirosis vaccine: immunogenicity and protective potential

**DOI:** 10.1007/s00253-024-13196-1

**Published:** 2024-07-22

**Authors:** Rafael Carracena de Souza Tapajóz, Francisco Denis Souza Santos, Natasha Rodrigues de Oliveira, Mara Andrade Colares Maia, Amilton Clair Pinto Seixas Neto, Laura de Vargas Maiocchi, Pedro Henrique Filgueiras Coelho Souza, Thaís Larré Oliveira, Odir Antônio Dellagostin

**Affiliations:** 1 Biotechnology Center, Technological Development Center, Federal University of Pelotas, Pelotas, RS Brazil; 2 Department of Microbiology and Parasitology, Institute of Biology, Federal University of Pelotas, Pelotas, RS Brazil

**Keywords:** *Leptospira*, Chimeric protein, Recombinant subunit vaccine, Recombinant inactivated *E. coli* vaccine, Recombinant bacterin, Adjuvants

## Abstract

**Abstract:**

Leptospirosis, a neglected zoonotic disease, is caused by pathogenic spirochetes belonging to the genus *Leptospira* and has one of the highest morbidity and mortality rates worldwide. Vaccination stands out as one of the most effective preventive measures for susceptible populations. Within the outer membrane of *Leptospira* spp., we find the LIC12287, LIC11711, and LIC13259 lipoproteins. These are of interest due to their surface location and potential immunogenicity. Thorough examination revealed the conservation of these proteins among pathogenic *Leptospira* spp.; we mapped the distribution of T- and B-cell epitopes along their sequences and assessed the 3D structures of each protein. This information aided in selecting immunodominant regions for the development of a chimeric protein. Through gene synthesis, we successfully constructed a chimeric protein, which was subsequently expressed, purified, and characterized. Hamsters were immunized with the chimeric lipoprotein, formulated with adjuvants aluminum hydroxide, EMULSIGEN®-D, Sigma Adjuvant System®, and Montanide™ ISA206VG. Another group was vaccinated with an inactivated *Escherichia coli* bacterin expressing the chimeric protein. Following vaccination, hamsters were challenged with a virulent *L. interrogans* strain. Our evaluation of the humoral immune response revealed the production of IgG antibodies, detectable 28 days after the second dose, in contrast to pre-immune samples and control groups. This demonstrates the potential of the chimeric protein to elicit a robust humoral immune response; however, no protection against challenge was achieved. While this study provides valuable insights into the subject, further research is warranted to identify protective antigens that could be utilized in the development of a leptospirosis vaccine.

**Key points:**

• *Several T- and B-cell epitopes were identified in all the three proteins.*

• *Four different adjuvants were used in vaccine formulations.*

• *Immunization stimulated significant levels of IgG2/3 in vaccinated animals.*

**Supplementary Information:**

The online version contains supplementary material available at 10.1007/s00253-024-13196-1.

## Introduction

Leptospirosis, caused by pathogenic spirochetes from the *Leptospira* genus, is a neglected zoonotic infection with high global morbidity and mortality rates (Adler and de la Peña Moctezuma, 2010; Costa et al. 2015). This disease poses a significant public health concern, affecting both humans and various mammals, including wild and domesticated animals (Costa et al. 2015; Narkkul et al. 2021). In urban environments, rodents are the primary reservoir hosts for *Leptospira* spp., shedding the pathogen in their urine and contaminating the environment. Humans, on the other hand, are accidental hosts (Adler and de la Peña Moctezuma, 2010; Haake and Levett 2015). In humans, leptospirosis can manifest with symptoms similar to those of other febrile bacterial and viral infections, including fever, headache, and muscle pain. In severe cases, it can lead to acute kidney injury, jaundice, and pulmonary hemorrhagic syndrome, with the most severe form being Weil syndrome (McBride et al. 2005). Annually, over 1.03 million individuals contract the disease, resulting in approximately 59,000 fatalities, predominantly in tropical regions (Costa et al. 2015). Additionally, animal leptospirosis remains a concern, especially in livestock, where it can lead to economic losses due to reduced productivity. Companion animals like dogs and cats are also at risk (Adler and de la Peña Moctezuma, 2010; Ellis 2015; Dorsch et al. 2020). In production animals, the infection can adversely affect their health, causing reproductive problems such as infertility, abortion, stillbirths, and reduced milk production (Adler and de la Peña Moctezuma, 2010; Martins et al. 2012).

Vaccines represent one of the most recommended and straightforward preventive measures against leptospirosis in at-risk populations, including both humans and animals, as effectively preventing the disease without a vaccine can be challenging (Adler and de la Peña Moctezuma, 2010; Ellis 2015; Grassmann et al. 2017b). The vaccines available for leptospirosis, known as bacterins, consist of mono- or polyvalent cell suspensions containing inactivated whole cells of the pathogen (Adler and de la Peña Moctezuma, 2010). While bacterin vaccines provide a short-term immune response, their effectiveness primarily relies on stimulating opsonizing antibodies, mainly targeting the lipopolysaccharide (LPS). These antibodies offer specific protection against the serovars included in the vaccine formulation or closely related ones (Adler and de la Peña Moctezuma, 2010; Verma et al. 2013). Currently, within the *Leptospira* genus, there are 64 officially classified species. Among them, at least 17 are pathogenic, with over 300 distinct infecting serovars grouped into 24 serogroups (Adler and de la Peña Moctezuma, 2010; Picardeau 2017; Vincent et al. 2019). Consequently, studies using bacterins have reported varying levels of efficacy, often highlighting their inability to prevent renal colonization by *Leptospira* spp. (Suepaul et al. 2010; Adler 2015; Sonada et al. 2018; Oliveira et al. 2019). Therefore, efforts have been directed toward creating recombinant vaccines that rely on highly conserved antigens shared among pathogenic *Leptospira* spp. (Dellagostin et al. 2011, 2017; Grassmann et al. 2017b; Oliveira et al. 2019).

Reverse and structural vaccinology, using genome sequencing and bioinformatics, has successfully developed more effective vaccines (Rappuoli 2000; Donati and Rappuoli 2013; Dellagostin et al. 2017; Maia et al. 2022). Epitope design in vaccine development identifies regions that can boost vaccine immunogenicity (Dellagostin et al. 2011; Bettin et al. 2022; Hamed et al. 2023). Outer membrane proteins (OMPs), due to their potential role in host–pathogen interactions, are promising vaccine candidates, with various recombinant vaccines incorporating conserved OMPs being evaluated. Protection levels vary, influenced by factors like administration route, adjuvant choice, and the animal model (Dellagostin et al. 2011; Grassmann et al. 2017b; Teixeira et al. 2020; Dorneles et al. 2020). Therefore, novel vaccine antigens are needed, which are serovar-independent and can hinder the spread of pathogenic *Leptospira* spp. The LIC12287, LIC11711, and LIC13259 genes encode predicted lipoproteins on the outer membrane of *Leptospira* spp., capable of binding and interacting with host components. This suggests their role in initial adhesion, evasion, and immune invasion processes, potentially contributing to virulence (Murphy and Weaver 2017; Kochi et al. 2019).

A study by Teixeira et al. (2020) demonstrated that recombinant forms of three outer membrane proteins (OMPs), namely rLIC12287, rLIC11711, and rLIC13259, adsorbed onto aluminum hydroxide (Al(OH)_3_), can induce a robust immune response against leptospirosis in immunized animals, resulting in 100% survival rates. In the present study, the objectives were to determine the conservation of OMPs (LIC12287, LIC11711, and LIC13259) among pathogenic *Leptospira* spp., predict surface-related T- and B-cell epitopes for these proteins, construct a synthetic gene encoding antigenic segments of these OMPs, express, purify, and characterize the resulting chimeric protein. The study aimed to evaluate the humoral immune response and immunoprotective potential in hamsters vaccinated with subunit vaccines using different adjuvants and an inactivated *Escherichia coli* bacterin expressing this chimera LIC12287/LIC11711/LIC13259 and characterize the IgG isotype profile in vaccinated animals.

## Materials and methods

### Bacterial strains and culture conditions

*L. interrogans* serovar Copenhageni Fiocruz L1–130 strain was cultured in Ellinghausen-McCullough-Johnson-Harris (EMJH) liquid medium supplemented with 10% *Leptospira* EMJH enrichment medium and 200 µg/mL of 5-fluorouracil at 30 °C without stirring. *E. coli* BL21 (DE3) STAR was cultivated in Luria–Bertani broth (LB) under agitation or LB agar, supplemented with 50 µg/mL kanamycin for selection when necessary, and incubated at 37 °C for 16–18 h.

### Sequence conservation among orthologs

Orthologous identification in leptospiral genome sequences from 63 *Leptospira* spp. with complete genome status was performed as previously described (Grassmann et al. 2017a), using the reciprocal best hit (RBH) method based on protein BLAST (BLASTp) searches (Camacho et al. 2009). *L. interrogans* serovar Copenhageni strain L1–130 proteins were used as the query sequences (GenBank accession numbers: AAS70858.1, AAS70300.1, and AAS71803.1). Protein sequences with > 70% similarity and > 40% coverage that were also the best reciprocal hit were considered conserved.

### Structural modeling and epitope predictions

Signal peptides were predicted using SignalP v. 4.1 (Almagro Armenteros et al. 2019) and removed from protein sequences for further analysis. The three-dimensional (3D) structures of each protein were predicted by protein threading modeling using the I-TASSER server (Zhang 2008). Structure refinement was performed using ModRefiner (Xu and Zhang 2011). RAMPAGE (http://mordred.bioc.cam.ac.uk/~rapper/rampage.php) was used to analyze the stereochemistry of each refined model by evaluating the Ramachandran plot server (https://zlab.umassmed.edu/bu/rama/) for each protein structure model. 3D structures were visualized using UCSF Chimera software v. 1.11.2 (Pettersen et al. 2004). B-cell and MHC-II epitopes were identified by BepiPred 2.0 (Jespersen et al. 2017) and NetMHCII 2.3 (Jensen et al. 2018), respectively. Epitopes were manually mapped onto the predicted 3D models of each protein in UCSF Chimera 1.12 in order to identify immunodominant regions. Predictions of physiochemical properties, stability, and half-life upon expression in *E. coli* were calculated using the ProtParam tool at ExPASy (Gasteiger et al. 2005).

### Design and cloning

The predicted structural models of the three proteins were utilized for antigen design. Immunodominant regions of each protein, characterized by a high number of linear epitopes, were selected. A flexible linker, Gly4 × SerGly4 × , was inserted between each gene portion to facilitate the proper folding of the protein (see Online Resource 1). The ProtParam tool and I-TASSER were employed to identify the best combination of selected fragments. The recombinant construct with the most favorable physicochemical properties (stability, isoelectric point, and half-life upon expression in *E. coli*) and structural model was chosen. A DNA coding sequence was chemically synthesized (GenOne Biotechnologies, Brazil), with codon optimization for *E. coli* expression, and provided in the pET28a vector. The sequences of recombinant chimeric construction (LIC12287/LIC11711/LIC13259 chimera) used in this study have been deposited in the National Center for Biotechnology Information (NCBI) gene bank (GenBank), accession number: OR988046.1 (see Online Resource 2).

### Expression, purification, and characterization of recombinant 6 × His-tagged chimeric protein

The gene for the chimeric construct (LIC12287-Linker-LIC11711-Linker-LIC13259) was cloned without signal peptide sequence. The chimeric protein was expressed and purified following a protocol previously described by da Cunha et al. (2019). *E. coli* BL21 (DE3) STAR cells were transformed with the pET28a vector containing the chimera gene through the heat shock method. The transformed cells were cultivated in LB broth with kanamycin (50 µg/mL) and 1 M glucose for 16–18 h at 37 °C and 180 rpm. When the culture reached an optical density of 0.6–0.8 at 600 nm, it was induced with 0.5 mM of isopropyl β-d-thiogalactopyranoside (IPTG) and incubated for an additional 3 h. Following induction, the cultures were centrifuged, washed with sterile saline solution (PBS), sonicated, and the insoluble fraction was suspended in a urea-containing buffer for 16–18 h. The protein was then purified using nickel (Ni2 +) affinity chromatography on a sepharose column with the ÄKTA™ Start system. The protein underwent urea dialysis in PBS buffer with Triton X-100 until a molarity of 3 M was reached. Purity and concentration were determined through electrophoresis, Coomassie Blue staining, *Western blotting* (WB), and quantification using the Pierce™ BCA Protein Assay Kit. The protein samples were stored at − 20 °C for subsequent preparation of subunit vaccines.

### Production of recombinant inactivated bacteria

For preparing recombinant *E. coli* bacterin and a negative control bacterin, *E. coli* BL21 (DE3) STAR cells were transformed using the heat shock method and the vectors pET28a/chimera and pET28a (negative control). These cultures were suspended in PBS, and serial dilutions were made from each cell suspension, which were then plated on LB agar containing kanamycin (50 µg/mL) and incubated at 37 °C for 16–18 h. The number of colonies on these plates allowed for the determination of viable cell numbers, adjusted to 10^9^ CFU/mL. Bacterial heat inactivation followed a protocol based on Sharma et al. (2011), with some adjustments. After inactivation at 80 °C for 30 min, samples were tested for growth by seeding them on LB agar and broth with or without kanamycin (50 µg/mL) and incubating at 37 °C for 168 h, assessing bacterial safety. Protein concentration in the extract was estimated through a 12% SDS-PAGE stained with Coomassie Blue and WB. Samples were then stored at − 20 °C for future use.

### Experimental vaccines formulation

To prepare intramuscular (IM) administered vaccines, two formulations were used: the pET28a/chimera recombinant bacterin and the pET28a bacterin, both containing 100 µg per dose. These were diluted in PBS and added to 15% (v/v) Al(OH)_3_ gel adjuvant (InvivoGen, USA). The mixture was gently mixed for 16–18 h at 4 °C, following the manufacturer’s recommendations. For the subunit recombinant vaccine, 100 µg of purified chimeric protein was prepared with 15% Al(OH)_3_ (v/v) in the same manner. For subcutaneously (SC) administered vaccines, the purified chimeric protein (100 µg per dose) was diluted in PBS and combined with different adjuvants: EMULSIGEN-D (MVP Adjuvants®, USA), EMULSIGEN-D with Al(OH)_3_, and Montanide™ ISA 206 VG (SEPPIC, France), adhering to the manufacturer’s guidelines. The ratio of adjuvant to total volume for EMULSIGEN-D was 20:80, and for ISA 206 VG, it was 50:50 (v/v). The EMULSIGEN-D/Al(OH)_3_ combination was prepared as previously described by Park et al. (2014), while the Sigma Adjuvant System (Sigma-Aldrich, USA) was used by diluting the chimeric protein in sterile saline solution and vortexing it to form the emulsion, with a final concentration of 2% oil. The stability of the experimental vaccines was assessed using the dropping method (McKercher and Graves 1976).

### Immunization and challenge of hamsters

Ten groups of 4-week-old female and male golden Syrian hamsters were organized, each containing ten animals. The groups received the vaccine and were monitored for immune response and protection against *Leptospira* infection. Groups 1 and 2 were administered intramuscular (IM) recombinant bacterin vaccines. Group 1 received the vaccine with Al(OH)_3_ adjuvant, while Group 2 served as a negative control and received a bacterin without the recombinant gene but with Al(OH)_3_. Group 3 received an IM recombinant subunit vaccine with Al(OH)_3_, and Group 4 received the same vaccine but subcutaneously (SC). Group 5 was a negative control for IM vaccines, receiving only PBS with 15% Al(OH)_3_. Groups 6 to 9 were immunized SC with recombinant subunit vaccines formulated with different adjuvants, including EMULSIGEN-D, EMULSIGEN-D + Al(OH)_3_, Sigma Adjuvant System, and ISA 206 VG, respectively. Group 10 served as a negative control for SC vaccines and received PBS only. All vaccinations consisted of two doses, 14 days apart. Blood samples were collected before and after vaccination; serum was separated and stored until analysis. On day 28 after the first dose, hamsters were challenged with 10 × endpoint dose (ED_50_), which corresponds to 10^4^ of *L. interrogans* serovar Copenhageni strain L1–130, with intraperitoneal inoculation. They were monitored for clinical signs of leptospirosis and euthanized if specific criteria were met, including 10% weight loss, prostration, and other symptoms.

### Evaluation of antibody response

To assess the humoral immune response, we employed an indirect enzyme-linked immunosorbent assay (ELISA) utilizing the chimeric protein. In brief, 96-well microtiter plates were coated with 50 ng of the antigen in 0.1 M carbonate-bicarbonate buffer at pH 9.6 and left at room temperature overnight. The blocking step was carried out using 5% powdered milk in PBS, and the plates were incubated for 1 at 37 °C. Serum samples were diluted 1:800 in PBS containing 2.5% milk powder and added to each well in triplicate. The plates were further incubated for 1 h at 37 °C. Peroxidase-conjugated anti-hamster IgG was applied, or anti-IgG subclasses (anti-IgG1, IgG2/3, and IgG3) were used, all diluted at 1:5000, followed by another 1-h incubation at 37 °C. Subsequently, the developing solution, consisting of 10 mL of phosphate-citrate buffer for substrate at pH 4.0, 4 mg of ortho-phenylenediamine (OPD), and 15 µL of H_2_O_2_, was added and allowed to react for 15 min in the dark at room temperature. To halt the reaction, 50 µL of stop solution, composed of 2N sulfuric acid (H_2_SO_4_), was added to each well. Absorbance measurements were obtained using a microplate reader with a 492-nm wavelength filter.

### Statistical analysis

Two-tailed Fisher’s exact test and Wilcoxon log-rank were used to define considerable differences in mortality and survival rates between groups. Analysis of variance was performed to compare the humoral immune response induced by different experimental groups (two-way ANOVA), followed by Tukey’s multiple comparison test; in view of that, there was a significant difference when *P* < 0.05. Graphic representations and statistical analyzes were performed using the GraphPad Prism 8 software.

## Results

### Conservation among the pathogenic *Leptospira* spp.

**Fig. 1:**
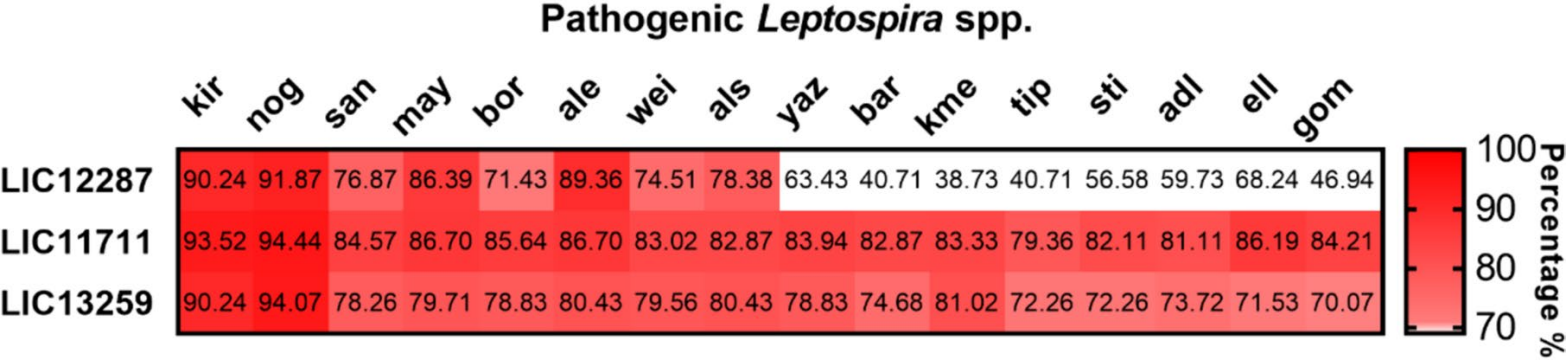
Conservation of the OMPs LIC12287, LIC11711, and LIC13259 from *L. interrogans* in 16 other pathogenic *Leptospira* spp. The sequences were compared using the RBH method based on BLASTp searches. Abbreviations: kir., *L. kirschneri*; nog., *L. noguchii*; san., *L. santarosai*; may., *L. mayottensis*; bor., *L. borgpetersenii*; ale., *L. alexanderi*; wei., *L. weilii*; als., *L. alstonii*; yaz., *L. yasudae*; bar., *L. barantonii*; kme., *L. kmetyi*; tip., *L. tipperaryensis*; sti., *L. stimsonii*; adl., *L. adleri*; ell., *L. ellisii*; gom., *L. gomenensis*

We screened the genome sequences of 63 *Leptospira* spp. to identify orthologous sequences. The proteins presented orthologs among the eight species that belong to node 1 in the P1 subclade of pathogenic *Leptospira* spp. (Vincent et al. 2019) with identities above 70%. All the pathogenic *Leptospira* spp. contained orthologs for the LIC11711 and LIC13259 proteins, with similarities ranging from 70.07 to 94.44% (Fig. 1), except for *L. kmetyi*, which did not contain an LIC13259 ortholog. LIC12287 had orthologs conserved only in the P1 subclade, considering only protein sequences with > 70% similarity and > 40% coverage, showing lower identity levels (ranging from 38.73 to 68.24%) in the other pathogenic leptospires, most of which were isolated from soil (Vincent et al. 2019). The three proteins were also absent from the saprophytic *Leptospira* spp. Among intermediate strains, only LIC11711 had orthologs with > 70% similarity (see Online Resource 1).

### Structural analysis and epitope prediction

Due to the importance of opsonophagocytosis in the clearance of leptospires during infection, we evaluated in silico the immunogenic potential of the selected OMPs to induce T- and B-cell responses. Overall, the three proteins showed epitopes for several HLA-DRB alleles evaluated distributed along the sequence. Additionally, multiple B-cell epitopes were identified in all three proteins. The list of surface-related T- and B-cell epitopes predicted for each OMP is provided in Online Resource 1.

### Recombinant protein construction

**Fig. 2:**
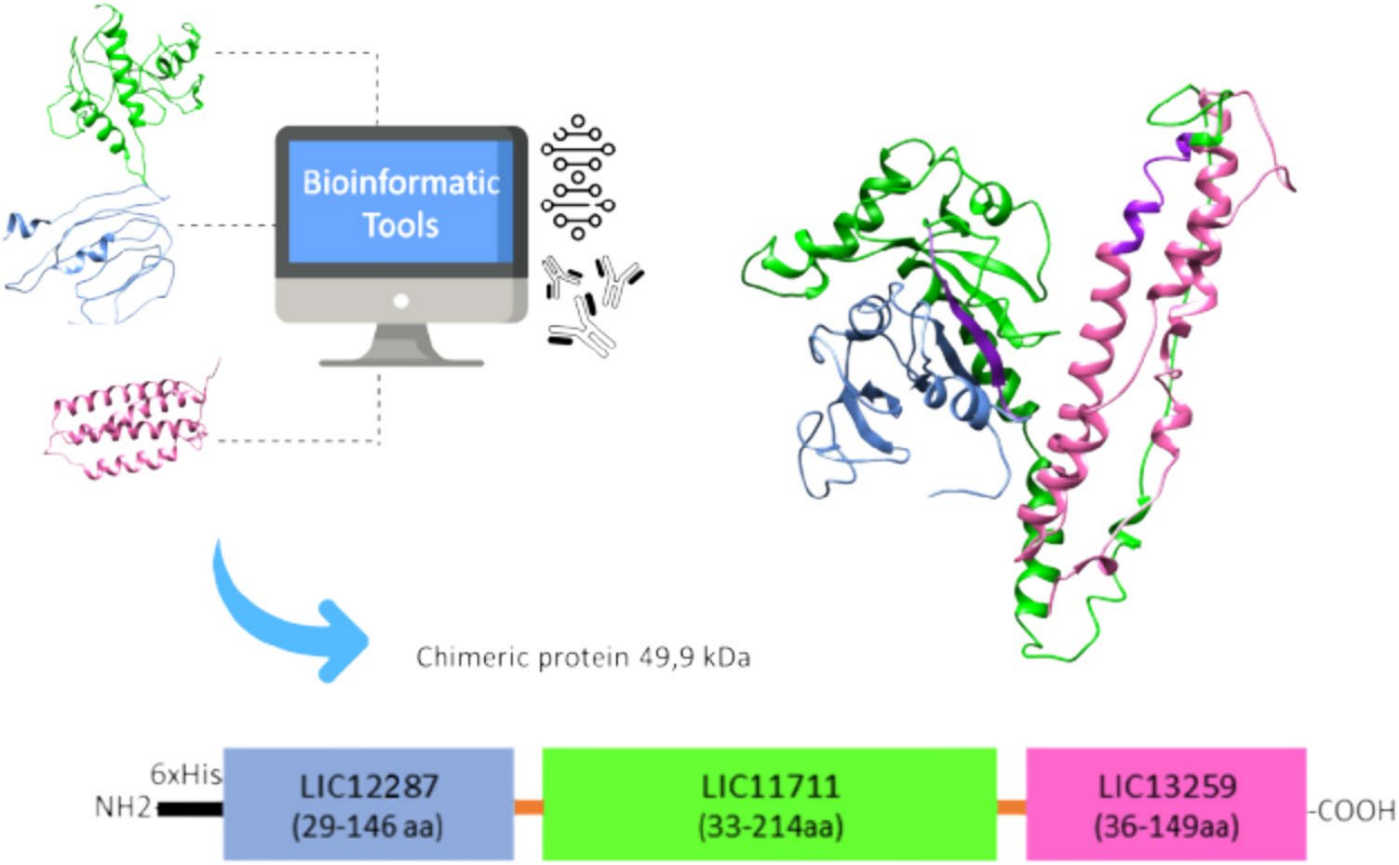
Schematic representation of the chimeric construction. Structural modeling of individual proteins LIC12287 (colored in blue), LIC11711 (colored in green), and LIC13259 (colored in pink) was performed using I-TASSER software. The 3D structure images were generated using UCSF Chimera software. Predicted B- and T-cell epitopes were mapped in each 3D model. Immunodominant regions as well as physicochemical parameters of each protein were considered to select the regions to compose the chimeric protein (indicated in parentheses). Each chimeric subunit protein is connected by a Gly4 × SerGly4 × flexible linker, presented in orange. The chimeric protein was fused to the 6 × histidine-tag of the pET28a vector at the *N*-terminal region

The characteristics of the antigenic sequences selected for constructing the chimeric protein are summarized in Table 1. Figure 2 displays the estimated in silico structures of each individual protein and the chimeric protein. To select immunodominant regions for chimeric construction, we considered the distribution of T and B epitopes mapped along the sequences and the 3D structures of each protein. We evaluated different orders of antigen combination. Physicochemical parameters analysis using ProtParam software for the best combination (LIC12287-Linker-LIC11711-Linker-LIC13259) indicated an estimated half-life of over 10 h in *E. coli*, classifying it as a stable. All three individual proteins, as well as the chimeric one, exhibited high TM-scores (> 0.5) and low RMSDs (< 2), indicating reliable and correct structural predictions. These results were further confirmed by the Ramachandran analysis, which revealed a low percentage of residues found at unfavorable angles (< 5%) (Table 1). A schematic representation of the immunodominant regions used to construct the synthetic gene is shown in Fig. 2.

**Table 1:** Quality assessment and structural models for the selected amino acid sequences of each individual protein and for the chimeric construct

Gene ID	Physicochemical parameters^a^					Structural parameters		
						I-TASSER model		Disallowed residues^d^ (%)
	MW	PI	II	Stability	*E. coli* half-life	TM-score^b^	RMSD^c^	
*LIC12287*	12.3	8.5	33.6	Stable	> 10 h	0.9691	0.808	0.000
*LIC11711*	20.7	6.4	29.3	Stable	> 10 h	0.9843	0.642	1.786
*LIC13259*	15.6	5.4	35.8	Stable	> 10 h	0.9637	1.104	0.813
Chimeric protein	49.9	6.9	34.6	Stable	> 10 h	0.9652	1.998	2.419

*MW* molecular weight, *PI* isoelectric point, *II* instability index

^a^Determined by ProtParam software

^b^TM-score: Template modeling score

^c^RMSD: Rootmean-square deviation of atomic position

^d^Percentual of disallowed residues determined by Ramachandran analysis

### Expression and characterization of the chimeric protein and vaccine production

**Fig. 3:**
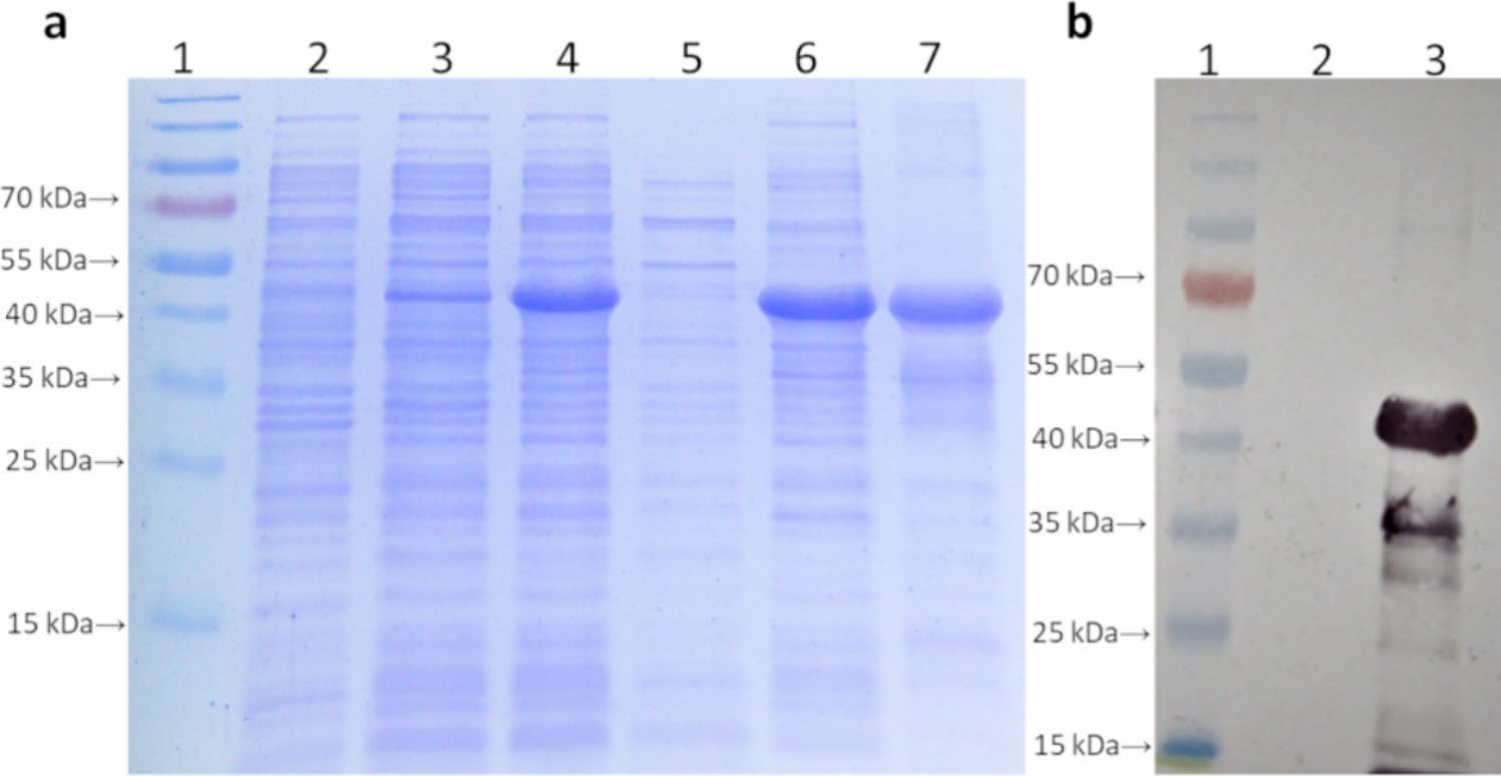
Characterization of the processes of expression and purification of the subunit chimeric protein. **a** Analysis on SDS-PAGE. 1, molecular weight marker; 2, negative control (*E. coli* STAR); 3, pre-induction chimeric protein; 4, post-induction chimeric protein; 5, soluble fraction chimeric protein; 6, insoluble fraction chimeric protein; 7, purified chimeric protein. **b** Analysis by WB, with peroxidase-conjugated anti-polyhistidine antibody. 1, molecular weight marker; 2, negative control (*E. coli* STAR); 3, purified chimeric protein

**Fig. 4:**
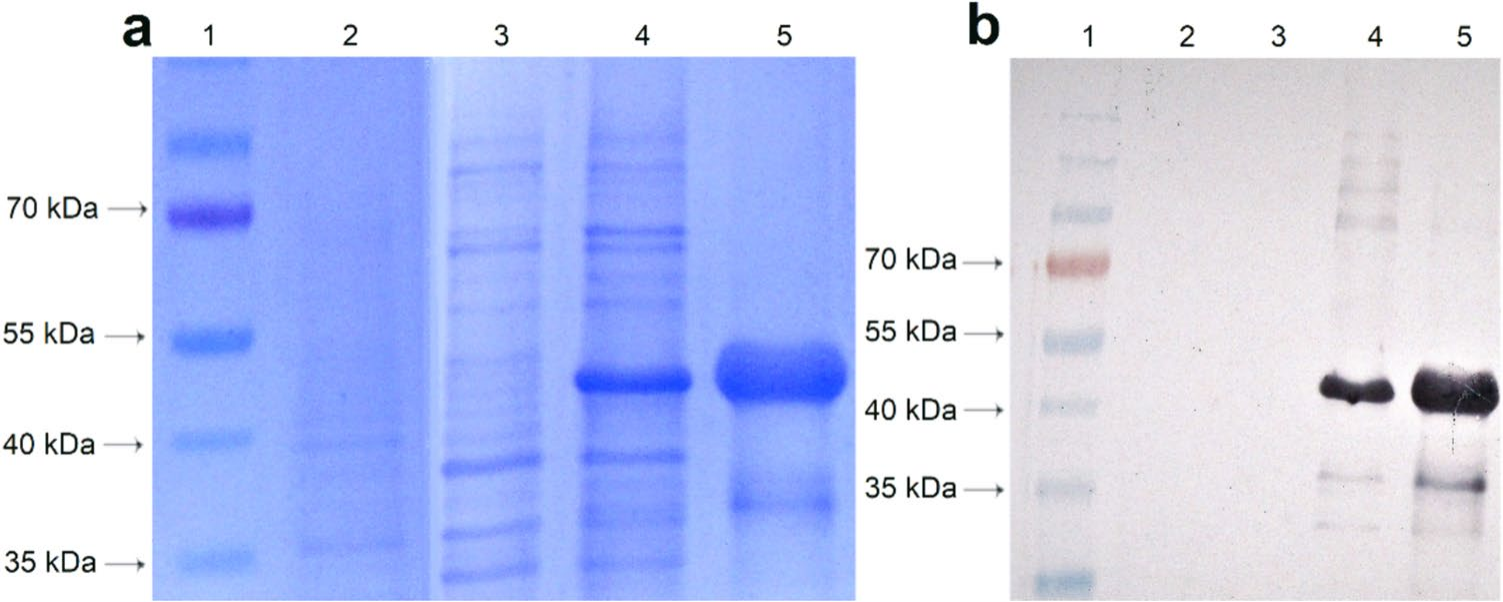
Characterization of the pET28a/chimera recombinant bacterin and pET28a bacterin (negative control) production processes. **a** Analysis on SDS-PAGE. 1, molecular weight marker; 2, negative control (*E. coli* STAR); 3, heat-inactivated bacterin pET28a; 4, heat-inactivated bacterin pET28a/chimera; 5, purified chimeric protein (positive control). **b** Analysis in WB, with peroxidase-conjugated anti-polyhistidine antibody. 1, molecular weight marker; 2, negative control (*E. coli* STAR); 3, heat-inactivated bacterin pET28a; 4, heat-inactivated bacterin pET28a/chimera; 5, purified chimeric protein (positive control)

Aliquots of the expression and purified protein processes were analyzed by SDS-PAGE (Fig. 3a) and WB using a His-tag antibody (Fig. 3b). The protein exhibited the expected size of approximately 49.9 kDa. Protein concentration was quantified, resulting in a concentration of 1.955 mg/mL. The characterization of the pET28a/chimera recombinant bacterin and the pET28a bacterin (used as a negative control) production processes can be observed in SDS-PAGE (Fig. 4a) and WB (Fig. 4b). The number of viable cells was adjusted to 10^9^ CFU per dose. Bacterin inactivation tests showed that CFUs did not grow over a period of 168 h, indicating the successful bacterial inactivation. Protein concentration in the bacterin was also quantified resulting in a concentration of 1.263 mg/mL.

### Antibody response in vaccinated hamsters

**Fig. 5:**
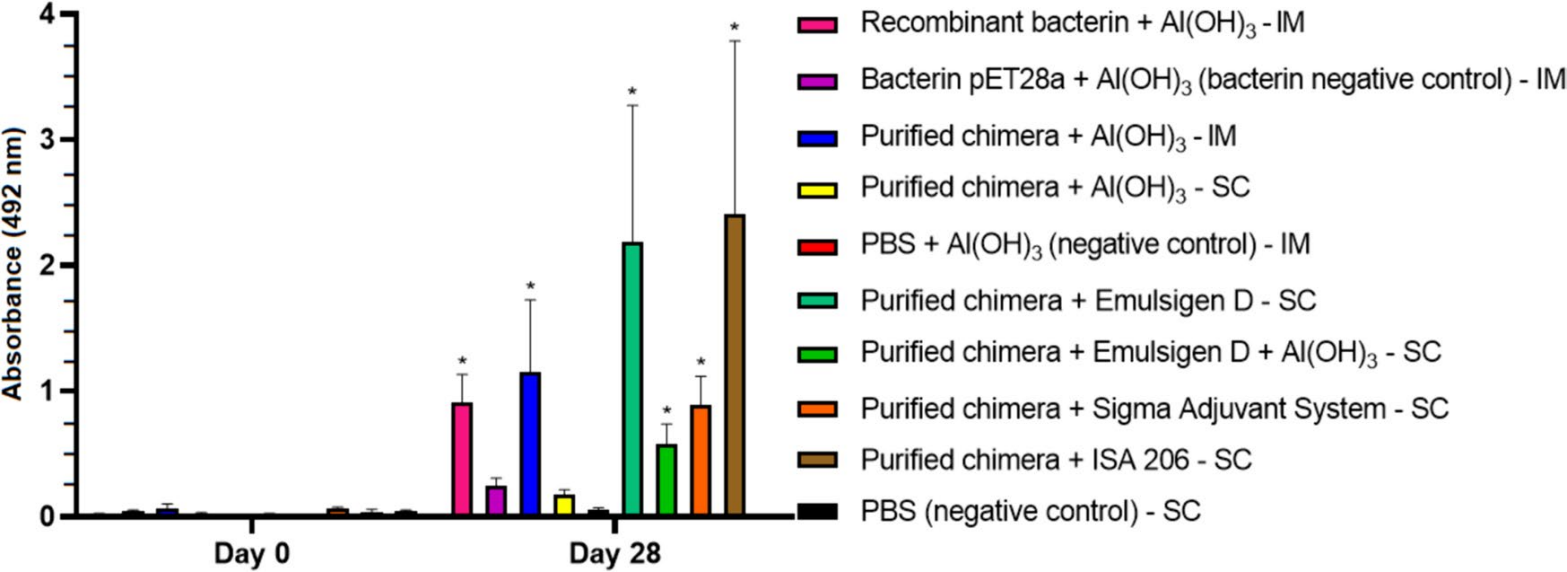
IgG antibody response in vaccinated hamsters with the chimeric protein evaluated by indirect ELISA, using serum samples collected on day 0 (pre-immune) and day 28 post-immunization, with the purified chimeric protein as antigen and a secondary anti-hamster IgG antibody. Results are presented as mean optical density (OD_492_) with standard deviation bars. Significant differences were determined by two-way ANOVA. An asterisk (*) indicates a significance difference (*P* < 0.05) between pre-immune sera and day 28 post-immunization. IM, intramuscular via; SC, subcutaneous via

**Fig. 6:**
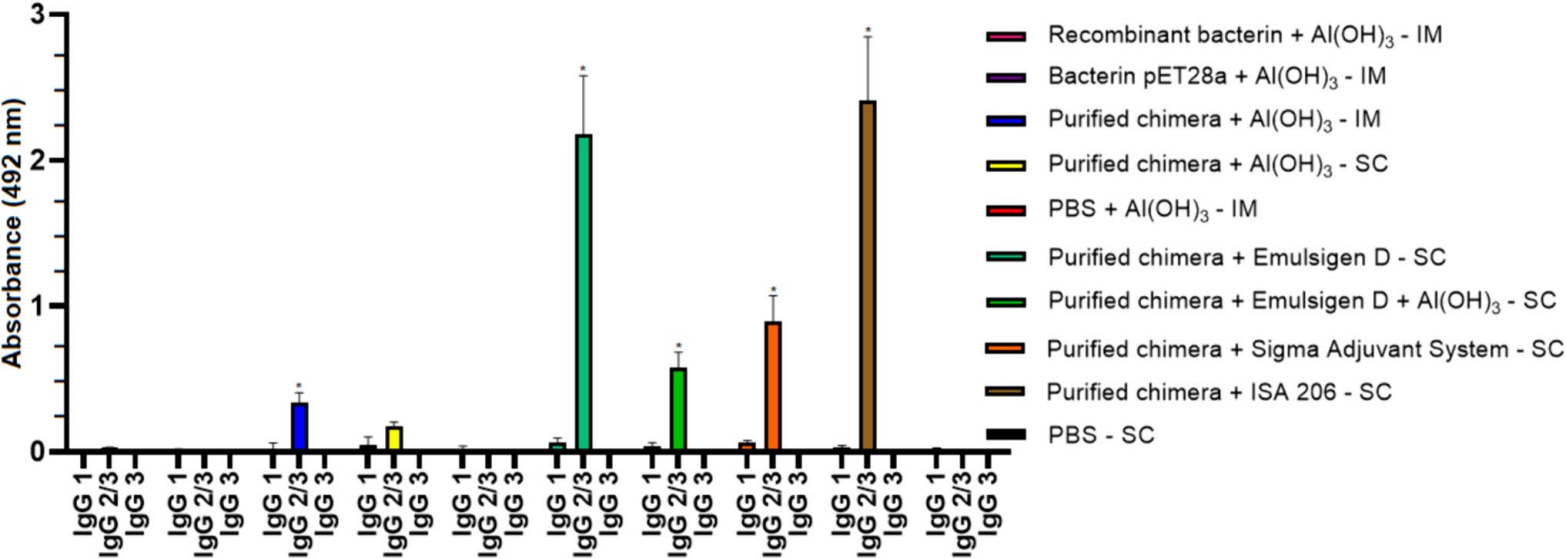
Evaluation of the IgG isotype subclasses IgG1, IgG2/3, and IgG3 produced after immunization with the chimeric protein. The results are presented as the mean optical density (OD_492_) with standard deviation bars. Significant differences were determined using a two-way ANOVA. An asterisk (*) indicates a significant difference (*P* < 0.05) compared to the control groups. IM, intramuscular via; SC, subcutaneous via

The antibody response in vaccinated hamsters was evaluated by indirect ELISA using serum samples collected on day 0 (pre-immune) and day 28 post-immunization, with the purified chimeric protein as the antigen and an anti-hamster IgG secondary antibody. Vaccinated hamsters produced an IgG response (*P* < 0.05), detected 28 days after the second dose when compared to the pre-immune samples and the control groups, demonstrating the potential of chimeric protein to promote a humoral immune response (Fig. 5). The only exception was the group vaccinated with purified chimera + Al(OH)_3_ by the SC route, which did not show significant difference. It is also worth noting that the animals immunized with the vaccine formulations with purified chimera induced antibodies against antigenic proteins at a statistically higher rate than animals immunized with the recombinant bacterin (*P* < 0.05). An analysis of IgG subclasses was performed on serum samples collected. Immunization stimulated significant levels of IgG2/3 in vaccinated animals (*P* < 0.05) (Fig. 6). The exceptions were the groups vaccinated with the recombinant bacterin + Al(OH)_3_ by the IM route and the purified chimera + Al(OH)_3_ by the SC route, which did not show significant difference. IgG1 and IgG3 isotype subclasses were not detected significant levels of antibodies.

### Efficacy of inactivated recombinant *E. coli* vaccine and recombinant subunit vaccines

**Fig. 7:**
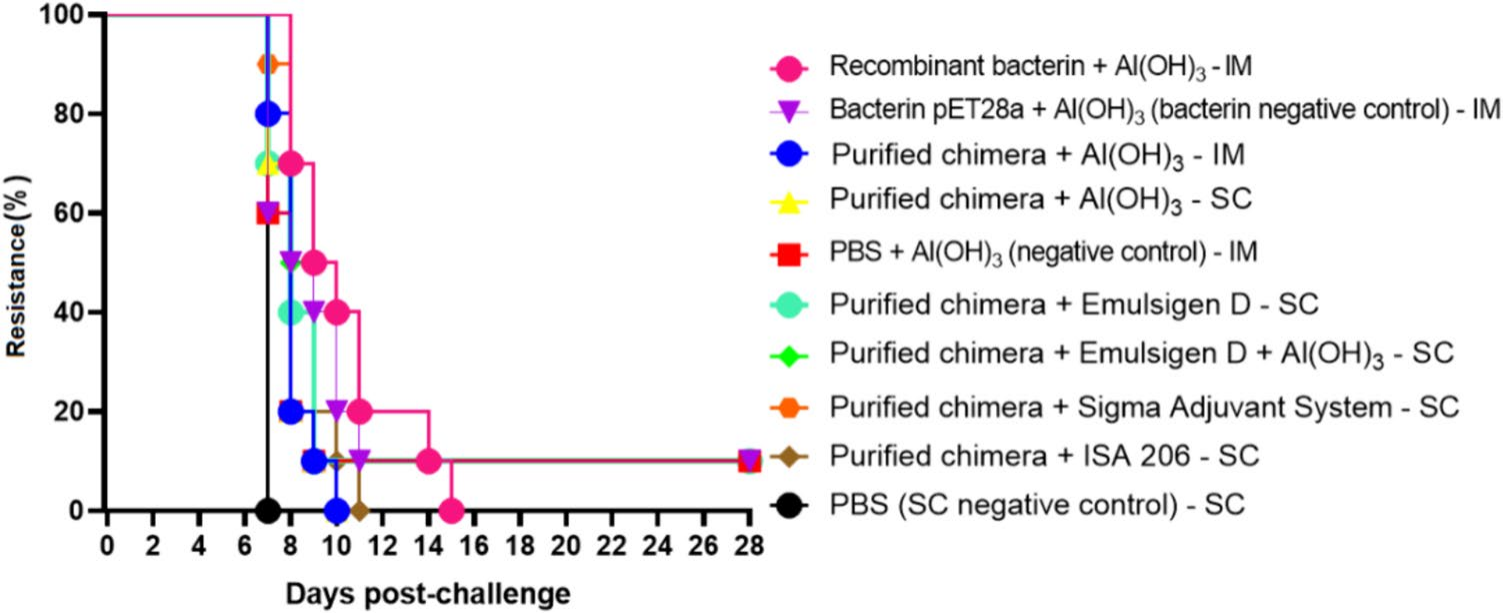
Protection against lethal challenge of acute leptospirosis in the hamster model elicited by immunization with inactivated recombinant *E. coli* vaccine (recombinant bacterin) and recombinant subunit vaccines containing the chimeric protein. Hamsters received two doses of 100 µL of vaccines with a 14-day interval. The routes of administration are indicated in the figure. On day 28 after the first dose, hamsters were challenged with an ED_50_ of 10.^4^ *L. interrogans* serovar Copenhageni strain L1–130 via intraperitoneal inoculation and monitored for an additional 28 days to assess clinical signs of leptospirosis. Endpoint criteria were observed in both the control and vaccinated groups between days 7 and 15 post-challenge. Only one animal from a vaccinated group and two animals from two of the control groups survived. No significant differences were observed (*P* > 0.05)

The inactivated recombinant *E. coli* vaccine (recombinant bacterin) failed to induce a significant protective immune response, resulting in 0% survival (see Fig. 7). Similarly, the recombinant subunit vaccines also proved ineffective in eliciting a substantial protective immune response. Notably, only one animal in the group immunized with the recombinant subunit vaccine, in combination with the adjuvant EMULSIGEN-D via the subcutaneous (SC) route, survived the lethal challenge posed by *Leptospira interrogans* (see Fig. 7). No significant differences were observed between the vaccinated groups and the control groups (*P* > 0.05). Endpoint criteria were met in both the control and vaccinated groups between days 7 and 15 post-challenge. As per the study protocol, these animals were promptly euthanized. The few survivors, including one from the vaccinated group and two from the control groups, were euthanized on day 28.

**Fig. 8:**
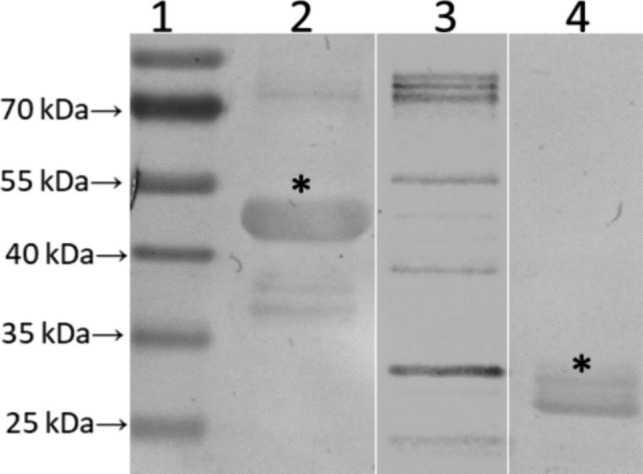
*Western blotting* analysis in which aliquots of the purified chimeric protein were subjected to SDS-PAGE and transferred to a nitrocellulose membrane. Serum from human naturally infected by *Leptospira* were used. 1, molecular weight marker; 2, purified chimeric protein; 3, negative control (*E. coli* STAR); 4, LipL32 (positive control). Asterisks in the second lanes indicate a detected protein band with an apparent molecular weight of approximately 49.9 kDa, likely corresponding to our purified recombinant chimeric protein and, 30 kDa for rLipL32

The recombinant chimeric protein was detected, through WB, by serum from humans naturally infected by *Leptospira* (Fig. 8). A protein band was detected in the second lane, with an apparent molecular weight of approximately 49.9 kDa, likely corresponding to the chimeric protein. As a positive control, another leptospiral recombinant protein, LipL32, was used and appeared in the fourth lane with its molecular weight of approximately 32 kDa (Fig. 8).

## Discussion

Developing an effective universal vaccine against leptospirosis poses a formidable challenge due to the extensive antigenic diversity within *Leptospira* spp. (Picardeau 2017). While recombinant outer membrane proteins (OMPs) show great potential as vaccine candidates, identifying the ideal candidate remains elusive (Teixeira et al. 2020). Leptospiral immunoglobulin-like proteins LigA and LigB have shown promise, yielding notable survival rates in animal models of leptospirosis (Conrad et al. 2017; Oliveira et al. 2019; da Cunha et al. 2019; Dorneles et al. 2020). Nevertheless, achieving sterilizing immunity remains a challenging goal, as demonstrated by the study conducted by Conrad et al. (2017). Furthermore, the limited conservation of the LigA protein among pathogenic *Leptospira* species underscores the pressing need to identify new conserved vaccine antigens. While numerous proteins have exhibited potential as vaccine candidates against leptospirosis (Dellagostin et al. 2017), it is paramount that selected proteins demonstrate robust conservation across pathogenic species to develop a universal vaccine (Maia et al. 2022). LIC12287, LIC11711, and LIC13259 are lipoproteins found on the outer membrane surface of *Leptospira* spp. They had previously demonstrated an ability to bind and interact with various host components, including elements of the terminal complement system pathway, suggesting their potential involvement in virulence (Figueredo et al. 2017; Cavenague et al. 2019; Kochi et al. 2019). Furthermore, each had previously been assessed for its capacity to induce an immune response capable of enhancing survival in hamsters challenged with virulent *Leptospira*. In our study, following in silico analysis, we constructed a chimeric protein composed of B- and T-cell epitopes from these three proteins. This chimeric protein was expressed in *E. coli*, characterized, and used to formulate several vaccine prototypes with different adjuvants, as well as a recombinant inactivated *E. coli* bacterin. The immune-protective potential of the vaccine prototypes was assessed in a hamster model of lethal leptospirosis.

In silico analysis revealed that the three proteins are highly conserved among pathogenic *Leptospira* spp. The bioinformatics tools used allowed the identification and selection of B- and T-cell epitopes for the construction of a stable chimeric protein incorporating these epitopes. B cells, as a crucial subset of lymphocytes, play a primary role in eliminating *Leptospira* by initially producing TLR4-dependent IgM antibodies that specifically target *Leptospira* LPS. Subsequently, they contribute to the immune defense by producing specific IgG antibodies (Werts 2010). Cellular immunity also holds significant importance in driving protective immunity against leptospirosis infections (Bolin and Alt 2001). More recently, Kumar et al. (2021; 2022) designed multiepitope-based vaccines with bioinformatics tools, from several immunogenic outer lipoproteins surface of *Leptospira*. Despite the promising potential shown in the immunoinformatic analysis, potency tests in experimental models should be performed to investigate whether these constructions are able to confer protection against lethal challenge. In this study, in order to obtain a better protection result, we first evaluated a chimeric protein based on immunodominant regions from three leptospiral lipoproteins. The next logical sequence would be to identify individual protective epitopes of each protein; however, in view of the negative results obtained here, this approach was not evaluated. In a research investigation, a multiepitope chimeric protein was formulated, comprising immunodominant epitopes from the OMPs OmpL1, LipL32, and LipL21, supplemented with Al (OH)_3_ as an adjuvant (Lin et al. 2016). In that study, the formulation provided 80% protection in a guinea pig model of leptospirosis. Furthermore, it triggered a cross-antibody response against various serogroups of *L. interrogans* (Lin et al. 2016). In another study, the multiepitope chimeric antigen based on LigA, Mce, Lsa45, OmpL1, and LipL41 proteins was tested associated with different adjuvants and was able to confer partial protection against infection in the leptospirosis in hamsters. Bettin et al. (2022) demonstrated that *Mycobacterium bovis* BCG strain expressing the chimeric antigen based in conserved surface exposed epitopes from three leptospiral TonB-dependent receptors: LIC10896, LIC10964, and LIC12374 induced a protective immune response in hamsters.

The efficacy of a vaccine hinges not only on its antigenic components but also on the adjuvants frequently employed to enhance immune system stimulation (Facciolà et al. 2022). While Al(OH)_3_ had been the sole adjuvant approved by the Food and Drug Administration (FDA) in the United States for use in commercially available human vaccines for many years (Murphy and Weaver 2017), other combinations of vaccines and adjuvants have been employed in the prevention of specific diseases (FDA 2019). In our study, apart from Al(OH)_3_, we employed three additional available adjuvant systems, namely EMULSIGEN®-D, Sigma Adjuvant System®, and Montanide™ ISA 206 VG. EMULSIGEN-D is an oil-in-water double adjuvant emulsion containing dimethyldioctadecylammonium bromide (DDA) in nanoparticles (MVP 2017). Cao et al. (2011) assessed innovative fusion proteins derived from extracellular matrix (ECM) binding domains of LigB, emulsified in EMULSIGEN-D, as potential vaccine candidates for leptospirosis in hamsters. The findings distinctly demonstrate that substantial antibody levels were produced after immunization with both native protein antigens and various fusion products. Survival data post-infection exhibited varying outcomes across different groups, offering protection ranging from 34 to 50% among the immunized animals (Cao et al. 2011).

Sigma Adjuvant System is a stable oil-in-water emulsion that serves as a viable alternative to traditional Freund’s water-in-oil emulsions. It is derived from components of bacterial and mycobacterial cell walls, delivering robust immune system stimulation (Sigma-Aldrich 2023). In a recent study conducted by Varma et al. (2022), the use of the AS04 adjuvant, Sigma-Aldrich’s adjuvant system, in LigA vaccines resulted in the generation of a notably enhanced immune response compared to Al(OH)_3_, with increased protective efficacy in a hamster model of leptospirosis. ISA 206 VG is a mineral oil-based adjuvant containing esters of octadecenoic acid and anhydromannitol in an oily solution and is used to formulate safe, stable, and water-in-oil-in-water emulsions (Seppic 2023). Balakrishnan and Roy (2014) conducted a comparative assessment of two experimental vaccines for bovine leptospirosis, both in laboratory and field settings. These vaccines were created using inactivated *Leptospira* serovars and were formulated with either ISA 206 VG or Al(OH)_3_ adjuvants. The response was notably higher in the ISA 206 VG vaccine when compared to the Al(OH)_3_ vaccine. The authors concluded that the enhanced effectiveness of the ISA 206 VG vaccine stemmed from the adjuvant, which induced a double emulsion effect, thereby extending the duration of immunity. In our study, while we did not observe a significant increase in hamster survival among those vaccinated with any of the vaccine formulations, hamsters that received SC vaccinations with our recombinant chimeric protein, in combination with the adjuvants ISA 206 VG and EMULSIGEN-D, exhibited the highest IgG antibody production. This was followed by hamsters vaccinated IM with the adjuvant Al(OH)_3_ and those vaccinated with the recombinant bacterin + Al(OH)_3_. Subsequently, hamsters vaccinated SC with the Sigma Adjuvant System and EMULSIGEN-D + Al(OH)_3_ displayed lower IgG production levels, respectively. Despite rLIC12287, rLIC11711, and rLIC13259 lipoproteins showed indices of protection in previous studies when evaluated individually (Teixeira et al. 2020), our chimeric construction failed to achieve protection in this study. The difference in results obtained by both research groups could be related to the protein dose used (50 µg/dose in Teixeira’s study versus 33 µg of each protein in the chimeric) and the presentation of epitopes in the protein folding. Although we used immunodominant regions of each protein, the arrangement of protective epitopes may have been compromised by the fusion of the proteins.

We assessed the immune response profile elicited by vaccination with the chimeric protein by examining IgG subclasses. It is worth noting that these different subclasses possess distinct effector functions, and in hamsters, IgG1, IgG2/3, and IgG3 subclasses are produced. It is suggested that elevated levels of IgG2/3 in hamsters are indicative of a Th1 immune response (Verma et al. 2015). In our investigation, the vaccine prototypes elicited notably significant IgG2/3 antibodies. Nonetheless, we were unable to establish a distinct correlation with the survival rate, as no statistical significance was observed in this regard. Teixeira et al. (2020), when using the individual proteins that comprise our chimera, also demonstrated high levels of IgG2/3. LIC12287, when administered alone, induced IgG2/3 responses, whereas LIC11711 and LIC13259 also elicited IgG1, albeit at lower levels. Furthermore, IgG1 and IgG2/3 responses were observed following a LigB subunit vaccine, which correlated with improved protection (Conrad et al. 2017). This suggests the possibility of multiple mechanisms of action that can effectively control *Leptospira* infection, particularly in the experimental hamster model (Teixeira et al. 2020).

Several studies have demonstrated promising outcomes utilizing *E. coli* bacterins expressing recombinant antigens. For instance, Moreira et al. (2016) focused on botulism, utilizing recombinant *E. coli* bacterins expressing botulinum neurotoxins (BoNT) to vaccine guinea pigs. They administered two subcutaneous doses and found that this approach eliminated the need for expensive protein purification processes, yet achieved results comparable to purified proteins, highlighting its potential for simplifying vaccine production in managing bovine botulism. Ferreira et al. (2018) explored the immunogenicity of a recombinant epsilon toxin (ETX) *E. coli* bacterin from *Clostridium perfringens* in rabbits and ruminants. They used unpurified recombinant proteins for subcutaneous vaccination, aiming to streamline recombinant vaccine manufacturing. The recombinant bacterin proved stable and consistent, inducing neutralizing antibodies against ETX. These findings underscore the potential of recombinant *E. coli* bacterins to simplify vaccine production while maintaining immunogenicity. Unfortunately, the *E. coli* bacterin expressing the chimeric antigen failed to provide any protection against challenge in the hamster model of leptospirosis.

In summary, our study assessed the humoral immune response and immunoprotective potential in hamsters vaccinated with a recombinant chimera consisting of lipoproteins in the form of a purified subunit vaccine with various available adjuvant systems, as well as a recombinant inactivated *E. coli* bacterin expressing this LIC12287/LIC11711/LIC13259 chimera. The analysis revealed significant IgG levels in vaccinated animals, indicating a robust specific humoral immune response to the selected epitopes and characterizing the predominant isotype as IgG2/3. Moreover, the chimeric protein was recognized by sera from human naturally infected by *Leptospira*, suggesting that LIC12287, LIC11711, and LIC13259 lipoproteins are expressed during infection in the host. However, none of the vaccine formulations induced a protective immune response in the hamster model following a homologous challenge with *L. interrogans*. Despite the rational antigen design and its incorporation into various vaccine strategies and formulations, the recombinant chimera, under these conditions, failed to confer protection against leptospirosis in hamsters. Further investigations are needed to deepen our understanding of the host immune response and enhance vaccine efficacy.

## Supplementary Information

Below is the link to the electronic supplementary material.

Supplementary file1 (XLSX 60 KB)

Supplementary file2 (PDF 290 KB)

## Data Availability

*Leptospira interrogans* serovar Copenhageni Fiocruz L1–130 strain is available as described in the Fundação Oswaldo Cruz (FIOCRUZ) *Leptospira* Collection (CLEP) (WDCM 1012). Any additional data and materials used in this article are available upon request.
