# Supplementary material for: Chimeric lipoproteins for leptospirosis vaccine: immunogenicity and protective potential
Source: Appl Microbiol Biotechnol. 2024 Jul 22;108(1):424. doi: 10.1007/s00253-024-13196-1 (PMC11263434; doi:10.1007/s00253-024-13196-1)
Supplement: Supplementary file 2 — Supplementary file2 (PDF 290 KB) [file 253_2024_13196_MOESM2_ESM.pdf]

Chimeric Lipoproteins for Leptospirosis Vaccine: Immunogenicity and Protective Potential

Affiliations

Rafael Carracena de Souza Tapajóz[1*]; Francisco Denis Souza Santos[1*]; Natasha Rodrigues de Oliveira[1]; Mara Andrade Colares Maia[1]; Amilton Clair Pinto Seixas Neto[2]; Laura de Vargas Maiocchi[1]; Pedro Henrique Filgueiras Coelho Souza[1]; Thaís Larré Oliveira[1]; Odir Antônio Dellagostin[1#]

[1] Federal University of Pelotas (Biotechnology Center, Technological Development Center), Pelotas (RS), Brazil.

[2] Federal University of Pelotas (Department of Microbiology and Parasitology, Institute of Biology), Pelotas (RS), Brazil.

[*] These authors contributed equally.

[#] Corresponding author: Odir Antônio Dellagostin – odirad@gmail.com

Online Resource 2. Sequences of recombinant chimeric construction used in this work.

LIC12287/LIC11711/LIC13259 chimera
GenBank accession number: OR988046.1

>LIC12287/LIC11711/LIC13259 chimera
gctagcggatccGGTCTGCGTCCAAACACGAACCCGACTAAAGACTATGGTTTCATGTACCCTATCATCTC
TAAAGGTGGCGTAATTGTTCACGATGGCGTTATTCCGGGTCCTCTGGGTGACAACGCGGAAAACAC
CTCTATCGGTAAAGCGTGCAGCAAAAATATCCTGTGGCTGGTCTCCTTCGGTGACTCTTCTATTGAG
GCAGCCAAAGCGGACGGTAAAATCACTAAAGTAGCAAGCATCGAATACGAGCAGCTGGGTGTACT
GTACGGTGCAATCTACCACAAATTCTGCACTGTAGTGACCGGTTCTAACGCGGCGGTTGAGACCAA
AACCGACGCGAAATCTGCTACCGGTAAAAAAGGTGGCGGTGGTTCTGGTGGCGGTGGTTCTAAGTC
TACCGATAACGAATCTATTAAATACGAATCTGTACCAAACCCTGCGGGCGAAAACGAAGTAGTTCT
GAACGAAGAAGGCCAGGAAACCACGCTGAATACCGGTGATCCGGCATCCTTCCTGAAACCGTCCA
AAGATCCGCTGGAATATTTCCGTGTGCACATCAGCTCTGACGGTTACCAGCTGCGTCAGCTGCGTG
GCAGCAAATTCATCAAACGTAAGGTAGATAAAGGTGGTGACGTGCTGATCAGCGAGGAACTGGCC
CGCTATAACAAAATTAACTTTATCGACGATGGTATTATCATCGTTGTGCTGAATGGTAACACTGGT
GCGTTCGAAACCATCCGCTTCAACACTCGTGTGCCGCGTATCAACGATCTGGCGAAAATCGTTCAA
AACGACGTTACCCGCTGGTCTATGGAACACAGCGAAGAAAAACCGGTAGTGACGAAGTTCCAAAT
CCACTACTCTCTGGAACTGAAAAACAAGACTGGCAGCACCCGTGATGCGGTTAAAGAAGAACTGA
AAAAGGAAGTCATTCGTGGTGGTGGTGGCTCTGGTGGTGGTGGTAGCGAACAGGAAAAAGATATT
CTGCAGCAGATTCTGATGGAAAATGAAAGCATCCACAAATTCCTGATGAAAGAAGAAGAGAAAAT
TCCGGACACTAACCAGCTGATTGCACACGTTGTTGAACTGGTTTCTCTGAACGGTGGTCTGAAATC
CCAGGCGGAGAAGATGCAGAACAGCCTGAAAGACAAAGAAACCCAGGACGTTGAAAAATTTTTCC
AGGCGTACTCCAGCTTTTCCGAAAACCTGGCGGAATCTCTGAAACTGGCGGGTGGCACCGGTGTTT
TCAACCGCTTCTATTGCCCGATGGTTAACAAAACTTGGGTTAGCCAAGGCACGAAAATTCGTtaataag
aattc

>translation (ExPASy)
ASGSGLRPNTNPTKDYGFMYPIISKGGVIVHDGVIPGPLGDNAENTSIGKACSKNILWLVSFGDSSIEAA
KADGKITKVASIEYEQLGVLYGAIYHKFCTVVTGSNAAVETKTDAKSATGKKGGGGSGGGGSKSTDNE
SIKYESVPNPAGENEVVLNEEGQETTLNTGDPASFLKPSKDPLEYFRVHISSDGYQLRQLRGSKFIKRKV
DKGGDVLISEELARYNKINFIDDGIIIVVLNGNTGAFETIRFNTRVPRINDLAKIVQNDVTRWSMEHSEEK
PVVTKFQIHYSLELKNKTGSTRDAVKEELKKEVIRGGGGSGGGGSEQEKDILQQILMENESIHKFLMKE
EEKIPDTNQLIAHVVELVSLNGGLKSQAEKMQNSLKDKETQDVEKFFQAYSSFSENLAESLKLAGGTGV
FNRFYCPMVNKTWVSQGTKIR--EF
